# Neonatal SARS-CoV-2 Infection: Practical Tips

**DOI:** 10.3390/pathogens10050611

**Published:** 2021-05-17

**Authors:** Cinzia Auriti, Domenico Umberto De Rose, Vito Mondì, Ilaria Stolfi, Chryssoula Tzialla, on behalf of the Study Group of Neonatal Infectious Diseases

**Affiliations:** 1 Neonatal Intensive Care Unit, Medical and Surgical Department of Fetus, Newborn and Infant—“Bambino Gesù” Children’s Hospital IRCCS, 00165 Rome, Italy; cinzia.auriti@opbg.net; 2 Neonatology and Neonatal Intensive Care Unit, Policlinico Casilino Hospital, 00169 Rome, Italy; vmondi.polcas@eurosanita.it; 3 Department of Neonatology, Policlinico Umberto I, “Sapienza” University, 00161 Rome, Italy; ilaria.stolfi@gmail.com; 4 Neonatal Intensive Care Unit and Neonatal pathology Unit, Policlinico San Matteo IRCCS Foundation, 27100 Pavia, Italy; c.tzialla@smatteo.pv.it

**Keywords:** SARS-CoV-2, COVID-19, pregnant women, pregnancy, neonates, infants

## Abstract

The recent viral pandemic in Wuhan, Hubei, China has led to the identification of a new species of beta-coronavirus, able to infect humans, the 2019-nCoV, later named SARS-CoV-2. SARS-CoV-2 causes a clinical syndrome named COVID-19, which presents with a spectrum of symptoms ranging from mild upper respiratory tract infection to severe pneumonia, with acute respiratory distress syndrome and frequent death. All age groups are susceptible to the infection, but children, especially infants, seem to be partially spared, having a more favorable clinical course than other age groups. There is currently no clear evidence showing vertical transmission and intrauterine SARS-CoV-2 infection in fetuses of women developing COVID-19 pneumonia in late pregnancy, and even if transmission is possible, the SARS-CoV2 positivity of the mother does not require delivery by caesarean section, does not contraindicate the management of the infant in rooming-in and allows breastfeeding. This review provides an overview on the biology of the virus, on the pathogenesis of the infection, with particular attention to pregnancy and neonatal age, on the clinical presentation of infection in newborns and young infants and summarizes the international recommendations currently available on the clinical care of neonates with SARS-CoV2 infection or at risk of catching the virus. The main objective of the review is to provide an update especially focused to the clinical management of COVID-19 infection in the perinatal and neonatal age.

## 1. Introduction

*Coronaviridae* is a family of enveloped, positive-strand RNA viruses that include four genera: alpha-coronavirus (α-CoV), beta-coronavirus (β-CoV), gamma-coronavirus (γ-CoV) and delta-coronavirus (δ-CoV) [1]. Six species of human coronaviruses (HCoV) were previously known. Four of them produce mild respiratory symptoms: HCoV-229E and HCoV-NL63 belong to α-CoV genus and HCoV-OC43 an HCoV-HKU1 belong to β-CoV genus [2,3]. Other two β-coronaviruses (severe acute respiratory syndrome coronavirus—SARS-CoV—and Middle East respiratory syndrome coronavirus—MERS-CoV), that moved from animal reservoirs, have been identified as causing agents of severe respiratory outbreaks.

Severe acute respiratory syndrome coronavirus 2 (SARS-CoV-2) is the seventh coronavirus that is contagious in humans. In December 2019, it was identified in China as the causing agent of coronavirus disease 2019 (COVID-19). The World Health Organization (WHO) declared this outbreak as a pandemic in March 2020. SARS-CoV-2 is a new β-CoV, associated with high contagiousness and fatality rate, up to 2% in infected subjects. Human-to-human transmission occurs primarily via respiratory droplets within a range of about 1.8 m [4], but can also occur through close contact, the fecal–oral route and ocular surface [5,6].

The virus has been identified using reverse transcription polymerase chain reaction (RT-PCR) in bronchoalveolar lavage fluid, saliva, and particularly in the nasopharyngeal swab of infected patients. The incubation period can vary between 2 and 14 days, being in most cases 3–7 days. SARS-CoV-2 primarily affects the respiratory tract, but potentially involves all organs and systems, including the placenta.

**Figure 1:**
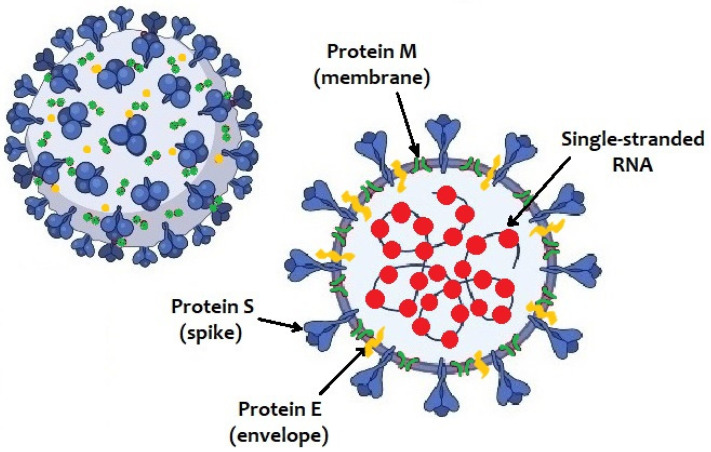
Structure of SARS-CoV-2 virus.

The SARS-CoV-2 genome consists in a positive-sense single-stranded RNA. Like other coronaviruses, it has four structural proteins: spike protein (S), membrane protein (M), nucleocapsid protein (N) and envelope protein (E) (Figure 1).

**Figure 2:**
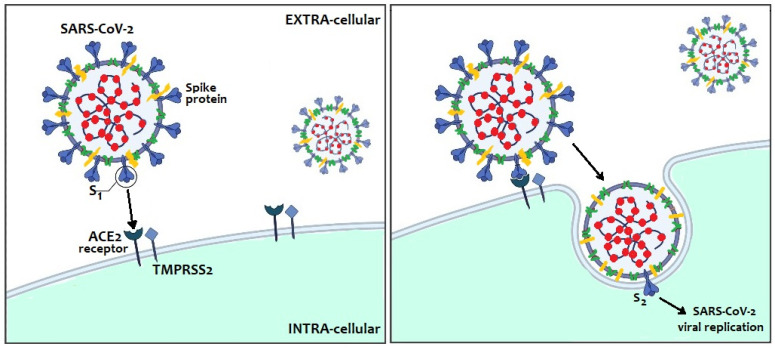
SARS-CoV-2 entry into host cells through its receptor-binding domain (RBD) and its affinity to human receptor ACE2.

Protein S plays a key role in the pathogenesis of infection in humans since it allows to the virus to anchor itself to the host receptors and entry into the host cells. It has two major subunits, S1 and S2. The distal S1 subunit, with two structural domains (the receptor-binding domain—RBD—and the N-terminal galectin-like domain—S1-NTD), has a role in receptor recognition and binding to the angiotensin-converting enzyme 2 (ACE2) receptor of host cells, while the membrane-anchored S2 subunit mediates the fusion of the viral and the host cell membranes [7] (Figure 2).

SARS-CoV-2 infects humans through binding between the RBD-domain of protein S and human receptor ACE2, widely expressed in many human cells, such as those of the respiratory tract, gut, uterus, ovaries and placenta [8,9]. Furthermore, protein S must be cleaved by certain host proteases, transmembrane peptidase/serine subfamily member 2 (TMPRSS2), as well as other lysosomal peptidases (e.g., cathepsins L and B), to maintain its infectious capacity and lead to virion entry and fusion into the human cells.

This review provides an overview on the pathogenesis of the SARS-CoV2 infection, with particular attention to pregnancy and neonatal age, on the clinical presentation of the infection in newborns and young infants and summarizes the international recommendations currently available on the management of neonates with SARS-CoV2 infection or at risk of catching the virus. The main objective of the review is to provide an update and practical tips especially focused on COVID-19 infection in the perinatal and neonatal age.

## 2. Methods

The literature search for this article was primarily Internet-based, using PubMed and Google. A list of search terms and phrases was compiled to focus on the general topics of ‘neonates’ or ‘newborns’ and ‘COVID-19’ or ‘2019-nCoV’ or ‘SARS-CoV-2’, without imposing restrictions on date or year, locations, study design, study aim, or inclusion/exclusion criteria. We reviewed all the articles published until 1st April 2021. Studies not written in English or Italian were withdrawn.

## 3. Transplacental Transmission of SARS-CoV-2

Despite the growing body of literature about the current SARS-CoV-2 pandemic, the impact of the virus contracted during pregnancy on the mother and fetus is still to be determined [10]. In past SARS and influenza H1N1 pandemics, pregnant women appeared to be more susceptible to infection in its most serious clinical forms, with a mortality much higher than that observed in the general population [11]. Available data on SARS-CoV-2 infection in pregnancy during the current pandemic are yet to be interpreted. It has been reported that pregnant women would be partially spared from infections with a more severe course, although pathophysiological changes associated with pregnancy (elevation of the diaphragm, increased oxygen consumption, impaired immune response) makes this category of patients more susceptible to more serious respiratory infections. In addition, infants of women with a SARS-CoV-2-positive placenta rarely manifest the disease, as later described. How the placenta enacts this protective effect on the fetus remains to be explained. Human receptor ACE2, which carries the virus into the host cells, is widely expressed in the placenta. In particular, ACE2 has been identified in the villi (syncytiothrophoblasts, cytotrophoblasts, vascular endothelium of the villi, smooth muscle of the primary villi), in the extra-villous trophoblasts, and in the decidua cells. Its expression increases along with the trimester of pregnancy, being able to transfer the virus transplacentally to the fetus much more during the later stage of gestation [12]. This would justify the lack of reports describing negative outcomes of maternal infection in the early stages of gestation, although we cannot yet exclude that they exist. Serine-protease type 2 (TMPRSS2) also found a similar dynamic alteration. Furthermore, the co-expression of serine protease type 2 (TMPRSS2) can promote entry by stimulating the fusion of viral particles with the host cell membrane and the related viral replication. Similar to other infections due to RNA viruses in pregnancy, SARS-CoV-2 infection causes placental lesions with signs of fetal and/or maternal vascular malperfusion and signs of inflammation. However, almost all infants of infected mothers were found to be negative at birth by RT-PCR and asymptomatic. Furthermore, it has been observed that the viral load in the placenta can be at least twice that of maternal blood and nasopharynx, increasing the possible risk of intrapartum transmission [13,14]. The theoretical risk of vertical transmission therefore has a biological plausibility and can occur both through the hematogenous route and, more rarely, by the ascending route [15].

A great caution is required in interpreting these data, because a complete collection from all tissues of both mother and baby is not always possible at appropriate times; currently available data often derive from case reports or case series rather than from multicentric studies. Regarding the unfavorable fetal/neonatal outcomes of COVID-19 infection contracted during pregnancy and perinatal outcomes, only four cases of miscarriage or abortion (1.4%) were reported [16]. No confirmed teratogenic effects were reported in cases where the infection was contracted early in gestation and the overall malformations’ rate in SARS-CoV-2-infected women was similar to that in uninfected women, according to data from a Spanish multicenter study [17].

In conclusion, transplacental transmission is possible or more likely in the last weeks of pregnancy. Vivanti’s recent report appears to have demonstrated neonatal viraemia following placental infection: the placenta showed signs of acute and chronic intervillous inflammation consistent with the severe systemic maternal inflammatory state triggered by SARS-CoV-2 infection. Both maternal and neonatal blood samples were positive, and the neonate had symptoms similar to those of infected adults [18,19].

## 4. How to Diagnose a Congenital Infection

The transplacental transmission of SARS-CoV-2 has a biological plausibility, although the data available to date must be interpreted with caution. Elevated specific immunoglobulin M (IgM) at 2 h of age were reported in a neonate born by caesarean section who was promptly separated and isolated from the mother at birth [20] and in a further three neonates with high IgM levels and with negative swabs [21]. Since IgM do not cross the placenta, we can hypothesize their fetal origin during intrauterine infection. In a recent meta-analysis that included 176 cases of neonatal SARS-CoV-2 infection, 5.7% of cases were classified as confirmed congenital infection [22].

Recently, a multidisciplinary group of experts from the World Health Organization (WHO) proposed a classification system to best define the vertical transmission of SARS-CoV-2, to allow for the comparison of data from different studies and to understand the clinical consequences for neonates born to infected mothers [23]. In relation to the timing of vertical transmission (in utero, intrapartum and early postnatal) four possibilities were categorized (Table 1): (a) confirmed; (b) possible (the evidence is suggestive but not confirmed for infection); (c) unlikely (poor support for diagnosis but infection cannot be completely ruled out); (d) indeterminate (when the tests required to define the classification have not been performed).

In utero infection can be defined as confirmed only if there is (1) “evidence of maternal infection” at any time during pregnancy and (2) “fetal exposure in utero” (when at least one neonatal specimen is positive for SARS-CoV-2 by 24 h of age) and (3) “SARS-CoV-2 persistence or immune response in the newborn” (at least one neonatal specimen was again positive at 24–48 h of age).

The presence of the viral persistence/immune response criterion in the newborn after 24 h of life is essential because a single positive RT-PCR datum, obtained too early on a neonatal nasopharyngeal sample, can indicate active viral replication, but also viral fragments acquired intrapartum or postpartum, or just a contamination.

For intrapartum and postnatal transmission, the maternal infection must be close to delivery (from 14 days before to 2 days after birth) (Table 2).

Most infants with suspected congenital infection were asymptomatic or paucisymptomatic: in a meta-analysis of 74 studies, 45% of 176 infants with rt-RT-PCR of nasopharyngeal exudate and/or presence specific IgM were asymptomatic [22]. When present, the symptoms were characterized by low-grade fever, mild hypoxia, polypnea. Invasive ventilatory support was rarely required [24]. Preterm infants require closer observation, as they may present with more severe respiratory symptoms, lethargy, and dehydration [25]. The outcome of neonatal infection is positive in most cases. According to data of the Italian National Institute of Health (accessed on 29 March 2021), the mortality observed in the group “0–9 years” was 0.01% [26].

## 5. How to Diagnose a Postnatal Infection

Most neonatal SARS-CoV-2 infections are therefore acquired after birth by horizontal virus transmission from the mother, healthcare workers, or other family members (Table 3). The source of the infection is often difficult to assess.

The belief that infants and children were partially spared in the two SARS-CoV-2 pandemic waves observed to date could derive from a possible underdiagnosis of the infection in pediatric age, since the symptoms described are mostly very poor or limited: fever or low-grade fever, lethargy, runny nose, cough, tachypnea, mild dyspnea, vomiting, diarrhea, abdominal distension and refusal to eat (Table 4) [22]. Former preterm infants could require up to high-frequency oscillatory ventilation, nitric oxide, prone ventilation and inotropic support to maintain oxygenation and ventilation [27].

## 6. Which Tests Are to Be Used in the Neonate?

To date, the molecular detection of SARS-CoV-2 is considered the gold standard test to diagnose COVID-19. The technique is PCR (RT-PCR), that consists in two PCRs in sequence: first a rt-PCR and, subsequently, a real-time PCR for the detection of viral RNA. The test displays the presence of genetic fragments of the virus both in asymptomatic/paucisymptomatic subjects and in symptomatic subjects but does not assess whether the virus is in the process of replication.

The molecular test is performed on mucus and respiratory secretions, collected with a nasopharyngeal swab through the nostril and/or pharynx. The molecular technique converts the viral RNA (+) genome into the complementary single-stranded DNA (cDNA) sequence, using reverse transcriptase. Real-time PCR is performed on the obtained cDNA, which simultaneously amplifies and quantifies the available DNA, while maintaining the relative concentration ratios of the viral RNA of SARS-CoV-2. For this reason, rt-real-time PCR is used in the diagnosis of COVID-19 (albeit with qualitative indications) [28]. 

Currently, the molecular test on nasopharyngeal swab is considered the most reliable for diagnosis, even in the newborn (with very high sensitivity and specificity), if the sampling technique and the analytical process are correct. A single positive RT-PCR result in a respiratory sample from a newborn can have several meanings: indicating active viral replication, or the presence of viral fragments acquired during passage through the birth canal or from external environmental contact soon after birth, or surface contamination that does not necessarily result in neonatal infection, symptomatic or not [29].

According to the before-mentioned WHO expert report, it appears that in the report of a universal nasopharyngeal RT-PCR screening for SARS-CoV-2, 2.2% (9/418) of neonates tested positive within 24 h of birth; of these, eight had mothers with negative RT-PCR on nasopharyngeal swab and without symptoms, and seven retested infants were negative on a second test [23]. This finding illustrates how complex it is sometimes to correctly interpret a positive molecular test in neonates. A negative test at 24 h from birth must be repeated, even on samples other than the nasopharyngeal, such as bronchoalveolar lavage fluid when indicated, blood and feces.

All neonates born to mothers with suspected or confirmed COVID-19 infection should undergo diagnostic rt-RT-PCR test on the respiratory tract exudate (nasopharynx, oropharynx, nose) at 24 h of life, regardless of the presence of symptoms in both the newborn and in the mother. A test performed too early may be indeterminate, but the best time to test the newborn is still unclear [23]. If the test is negative or indeterminate, it must be repeated at 48 h of life. Asymptomatic infants who can be discharged before 48 h after birth should be tested before discharge (at 24–48 h of age). There is no need to wait for the test result to discharge the neonate if he or she appears clinically stable.

Serological tests search for SARS-CoV-2-specific antibodies in serum. They should not be used for diagnostic purposes and do not replace the identification of viral genetic material from the nasopharyngeal swab. The use of serological tests aims to evaluate the progress of the infection in the individual and in the community. In the newborn, they are not indicated, and interpretation can be very difficult. In fact, during the second and third trimester of gestation, maternal immunoglobulin G (IgG) are transferred to the fetus through the placenta. The presence of IgG antibodies in the newborn at birth may simply reflect the transfer of maternal antibodies and does not allow the diagnosis of intrauterine infection. Maternal IgM and IgA do not cross the placenta unless there are lesions and are thought to represent the immune response of the fetus to intrauterine infection. However, the sensitivity and specificity of IgM tests vary and are usually lower and less reliable than the molecular test. Therefore, a positive serological test in a newborn at birth always requires confirmation with a molecular test. To date, the presence of positive SARS-CoV-2 IgM in a newborn between birth and 7 days of life suggests the suspicion of a fetal response to an intrauterine infection, while positive IgM after 7 days suggests an early intrapartum or postnatal infection [23].

During COVID-19 infection, there are no specific laboratory data in neonates and little infants. Leukopenia and lymphocytopenia have frequently been observed, while C-reactive protein and procalcitonin are usually normal. Other anomalies observed were mild to medium thrombocytopenia, an increase in creatine phosphokinase, alkaline phosphatase, alanine aminotransferase and lactic dehydrogenase. Lung lesions are better seen with pulmonary axial computed tomography rather than radiography alone and consist mostly of multiple radiopaque nodular images, single or multiple foci of parenchymal consolidation mostly located at the periphery of the lung parenchyma [30]. 

## 7. How to Treat SARS-CoV-2 Infection

The approach to SARS-CoV-2 infection depends on the patient’s symptoms. From the respiratory point of view, if respiratory support is required, the neonate born to a mother with suspected or confirmed SARS-CoV-2 infection should be managed with devices currently used in clinical practice, carefully limiting the generation of aerosols and the spreading of droplets exhaled during oxygen delivery and ventilation strategies. The risk of aerosol transmission from neonates with SARS-CoV-2 to healthcare workers during respiratory support may be low. However, further studies on viral dispersion with different-sized droplets and various types of neonatal respiratory support are needed [31]. Therefore, given the still not fully known characteristics of the current pandemic, it is prudent that health workers wear complete personal protective equipment during respiratory assistance to the newborn. The elective intubation of newborns born to SARS-CoV-2-positive women is not recommended and patient management must be tailored to the needs. When assembling the mechanical ventilator, it may be useful to place a hydrophobic antiviral/antibacterial filter on the expiratory branch of the circuit, paying attention to the effect of the increase in dead space in extremely preterm infants.

From the pharmacological point of view, most clinical trials did not include pregnant women with ongoing infection, although the safety of various drugs (i.e., lopinavir-ritonavir and remdesivir) during pregnancy has already been evaluated previously [32]. In neonatal age, no therapy is usually required in asymptomatic cases, while supportive therapy and paracetamol are recommended in mild–moderate cases. In severe cases (clinical worsening, respiratory distress, sepsis, shock), it is possible to start intravenous remdesivir at the loaded dose of 5 mg/kg/per day iv (in 30 min) on the first day, and of 2.5 mg/kg/per day in the following nine days. Note that the most appropriate dosage of remdesivir in the first 2 weeks of life and for babies with a body weight lower than 2.5 kg has not yet been established [33,34,35]. A higher-than-expected incidence of thromboembolic complications has been described when the clinical course of COVID-19 is severe. It may therefore be appropriate to start enoxaparin at a dose of 150–300 IU/kg/per day [36,37]. Sometimes, the cytokine storm in progress leads to COVID-19-associated multisystem inflammatory (MIS-C). In such a situation, it could be appropriate to start the immunomodulatory therapy using IV immunoglobulins (400 mg/kg/day for 5 days or 1 g/kg/day for 2 days) or monoclonal (anakinra or tocilizumab) [38,39,40].

## 8. Breastfeeding and SARS-CoV-2 Infection

SARS-CoV-2 virus has not been detected in human milk samples in most reports [41,42]. Although in the detection of SARS-CoV-2 RNA in some human milk samples, the risk of the milk contamination by maternal respiratory secretions could not be ruled out, as declared by the same authors [43]. Breastfeeding seemed to be not associated with SARS-CoV-2 infection, revealing that viral transmission through milk, if any, is very rare. A particular caution should be acknowledged when analyzing these results, considering the method of milk collection, sample handling and storage, validation of used assays, viral quantification and viability [42]. The presence of viral particles does not imply the possibility of transmitting the infection. Conversely, the benefits of breastfeeding concern the transfer of specific antibodies to the neonates, the quality of nutrition and prevention of obesity in late life, the development of the infant immunity by immune-stimulating factors and microorganisms colonizing the gastrointestinal tract [44].

Therefore, the current indications of all the Italian and foreign scientific societies and of the CDC invite mothers with suspected or confirmed COVID-19, if in good clinical condition, to breastfeed their babies, adequately applying all the correct hygiene rules (precautions from contact and droplets) [45,46]. Both parents must be informed of the remote possibility of transmission and an informed consent should be signed.

It is clear to date that milk produced by infected mothers is a source of anti-SARS-CoV-2 IgA and IgG and can neutralize SARS-CoV-2 activity, supporting the recommendations to continue breastfeeding although mild-to-moderate COVID-19 illness [47].

## 9. How to Rethink In-Hospital Management of Mother and Neonate

If the pregnant woman has a suspected or confirmed SARS-CoV-2 infection, a dedicated path must be provided within the hospital structure, as recommended [48,49]. Triage must be organized to separate infected patients from non-infected patients, placing them in dedicated rooms, where it is possible to assess them safely. Dedicated healthcare workers must be equipped with all the necessary personal protective equipment (PPE: gloves, surgical mask or FFP2 or FFP3, eye protection, disposable gown, overshoes). The patient must wear the mask from the moment of entry into the hospital. The birth must take place in a dedicated delivery room, with a multidisciplinary assistance team and with a minimum number of operators. The assistance and monitoring protocol is accorded to the routinary clinical practice of the unit, adapted to the clinical conditions of the pregnant woman, regarding caesarean section, continuous cardiotocography monitoring and steroid prophylaxis for fetal lung development. In symptomatic women, water birth is contraindicated. After the birth, the newborn is managed in the resuscitation unit, in the delivery room, at a distance of at least two meters from the mother, or in a dedicated room, next to the delivery room. Delayed cord clamping can be performed on a routine basis, depending on the condition of the newborn. Skin-to-skin contact, including kangaroo mother care for preterm or low birth weight infants, is recommended if the clinical conditions of the infant and mother allow it [48,49]. 

When transferred to the NICU, the infant should be placed in a closed transport incubator, with immediate disinfection immediately after the transport. The assistance in the unit must be provided in an area isolated from other patients, within which the medical and nursing staff act following all the needed precautions, minimizing any contact with the patient. By following the appropriate preventive measures, the parents’ entry to the unit must be guaranteed. After delivery, when the baby and mother leave the delivery room, all devices must be removed and placed in closed plastic bags, with adequate disinfection of the environment and equipment. If the clinical condition of the newborn is stable and the mother is paucisymptomatic, she can independently manage the newborn in rooming-in and they must be managed together, pending the results of any tests performed. If the mother’s test is positive, rooming-in in a dedicated and isolated room can be performed [50].

Infants born to SARS-CoV-2-positive mothers or infants whose mother’s test was pending should be considered persons under investigation (PUIs), even if PCR resulted negative at 24 h of life. Considering the incubation period (range, 2–14 days; mean 4–5 days), they should be retested to rule out the perinatal transmission of SARS-CoV-2 (due to contact with maternal secretions), before their status is resolved. The duration of transmission precautions has to be discussed on a case-by-case basis with an infection prevention and control team [51]. 

Mother–newborn separation should occur only in the case in which the conditions of the mother are severe, especially from the respiratory point of view. It is recommended to directly breastfeed the infant at the mother’s breast if the mother is in stable clinical conditions or to express milk if the mother cannot breastfeed the infant due to compromised clinical conditions. The following PPE is recommended for operators assisting the mother and the newborn: water-repellent surgical masks and (where available) FFP2, disposable gown, gloves and devices that allow eye protection, overshoes. When maneuvers that allow aerosol transmission are necessary (i.e., during intubation, if the patient is undergoing ventilatory assistance, during aerosol therapy, during bronchoscopy or during cardio-pulmonary resuscitation), healthcare workers should wear FFP2 and, where available, FFP3 masks [52].

Given the highly contagious nature of SARS-CoV-2, additional precautions should be considered during the transport of patients with suspected or confirmed COVID-19. Dedicated pathways have to be carefully planned. A recent European consensus recommended transporting the infant without parents or relatives, regardless of if they are symptomatic or not, to reduce the risk for the transport team of becoming infected during the transport [53].

Healthcare workers should assist the infant in a closed transport incubator, avoiding reopening the portholes, except in the case of resuscitation procedures. At the end of transport, any exposed equipment should be disposed (i.e., masks or self-inflating bag) or sterilized (i.e., laryngoscope) according to routine procedures [52]. All procedures must always be performed wearing disposable PPE. The vehicle requires decontamination with a universal detergent (i.e., ethanol) followed by cleaning of the entire interior of the vehicle with a chlorine-based solution at 1000 parts per million and waste should be disposed of as infectious waste, as per local policy [53].

## 10. Which Indications to Provide upon Discharge?

The clinical conditions of the mother and neonate seem the best indicator for hospital discharge of the mother–infant dyad. From the studies carried out to date, it seems that the concentration of SARS-CoV-2 RNA measured in the samples from the upper respiratory tract is reduced after the onset of symptoms in patients with mild-to-moderate forms [32]. The replicating virus was not found 10 days after the onset of the disease, although the absence of viral replication between 10 and 20 days after the onset of symptoms has been documented in immunocompromised individuals with serious clinical forms [54]. After discharge, the mother who has had a SARS-CoV-2 infection can stop her quarantine: (1) after 10 days from the onset of symptoms in asymptomatic or paucisymptomatic infections; (2) after 20 days in severe infections and in immunocompromised patients; (3) 24 h after the resolution of fever (without taking antipyretics) and other symptoms. Viral testing to discontinue isolation is no longer recommended unless immunocompromised [55]. Discharge of the neonate is not equally conditioned by the result of the nasopharyngeal swab test. The test result can be communicated to the family even after discharge. From a general point of view, the newborn can be discharged when he or she meets Benitz’s discharge criteria [56]. The criteria for interrupting the quarantine precautions of the neonate are: (1) at least 10 days elapsed since the onset of symptoms; (2) at least 24 h elapsed since the resolution of the fever without administration of antipyretic drugs; (3) improvement of all clinical symptoms. After discharge, it is important to schedule a follow-up appointment for the infant.

## 11. Vaccines and Preventive Strategies

At the time of writing, the available vaccines are giving hope for the prevention of the disease in adulthood. According to CDC interim guidelines, people are considered fully vaccinated for COVID-19 ≥2 weeks after they have received the second dose in a 2-dose series (Pfizer-BioNTech or Moderna), or ≥2 weeks after they have received a single-dose vaccine (Johnson and Johnson/Janssen) [57]. To date, vaccination for infants and children is not foreseen: the first reports about Pfizer-BioNTech administration to adolescents (12–15 years) seem to be promising, but data have not yet been reviewed by independent experts [58]. Unfortunately, the fight against COVID-19 is not over yet. The emergence of new variants with greater spreading capacity (such as B.1.1.7 from the UK, B.1.351 from South Africa and B.1.617 from India) is an expected event, considering that RNA viruses typically have rates higher mutation rates than DNA viruses. We will have to address this evolving situation with international surveillance, monitoring and containment strategies. Daily preventive actions to reduce the spread of COVID-19, including social distancing, protective masks and hand hygiene still remain the cornerstones of this fight. Our hope is that more and more vaccine doses will be available and administered in the shortest time frame and that current vaccines can provide effective sustained protection, beyond some changes in antigenic sites in the new SARS-CoV-2 variants.

Furthermore, in order to reduce the risk of transmitting the infection to neonates, the vaccination of pregnant women appears to be a safe strategy. The first data reported that a robust humoral immunity is generated in pregnant women after COVID-19 mRNA vaccines: vaccine-generated antibodies were present in all umbilical cord blood and breastmilk samples of their infants [59].

## 12. Strength, Limitations and Conclusions

In this review, we reported a full-range view of the perinatal and neonatal aspects of the COVID-19 infection, including a summary of the international indications currently available on the clinical management of patients and preventive strategies against the infection. The main limitation is the provided data derive from case series or studies concerning limited cohorts, as the published data regarding the national registers about neonatal SARS-CoV-2 infection are currently not available in the literature.

In summary, there is still no clear evidence showing vertical transmission and intrauterine SARS-CoV-2 infection in fetuses of women developing COVID-19 pneumonia in late pregnancy, even if transmission is possible. The mothers’ SARS-CoV2 positivity does not require delivery by caesarean section, does not contraindicate the rooming-in management of the infant, with precautions against transmission by contact route and allows breastfeeding. Mother–newborn separation should occur only in the case in which the conditions of the mother and/or of the neonate are severe, especially from the respiratory point of view.

Research perspectives regarding the pregnant mother and the newborn concern mainly the safety and efficacy of the administration of currently available vaccines during gestation, the persistence of immunity and the transferability to the fetus/newborn. Furthermore, there is much to understand about the infant’s resistance to infection and whether it actually exists.

## Figures and Tables

**Table 1:** Diagnosis of in utero SARS-CoV-2 infection requires three criteria: (1) evidence of maternal SARS-CoV-2 infection anytime during pregnancy AND (2) in utero fetal SARS-CoV-2 exposure (RT-PCR from a sterile sample, placental tissue, RT-PCR from a non-sterile sample, serology IgM and IgA) AND (3) SARS-CoV-2 persistence or immune response in the neonate (modified by WHO guidelines [23]).

IN UTERO Infection	(1) Maternal SARS-CoV-2 Infection	(2) Evidence of In Utero Fetal SARS-CoV-2 Exposure (Birth–24 h)	(3) Viral SARS-CoV-2 Persistence/Immune Response (24–48 h)
**Confirmed**	Suspect, probable or confirmed maternal infection **anytime during pregnancy**	**Yes**	**Positive RT-PCR ^1^**
**Possible**		**Yes**	**Positive RT-PCR ^2^** **OR** **Positive serology** (IgM or IgA) at age 24 h–<7 days
**Unlikely**		**Yes**	**Negative RT-PCR ^1,2^** **OR** **Negative serology** (IgM or IgA) at age 24 h–<7 days
		**No**	**Positive RT-PCR ^1,2^** **OR** **Positive serology** (IgM or IgA) at age 24 h–<7 days
**Indeterminate**		**Yes**	**No** tests (above) performed
		**No** in utero fetal exposure tests performed	**Positive RT-PCR ^1,2^** **OR** **Positive serology** (IgM or IgA) at age 24 h–<7 days

^1^**RT-PCR from sterile samples**: amniotic fluid (sterile collection caesarean section prior to rupture of membranes or amniocentesis), neonatal blood (cord blood needs confirmation with peripheral blood or other sample), lower respiratory tract samples obtained by bronchoscopic or non-bronchoscopic bronchoalveolar lavage, bronchial or tracheal aspirate, or cerebrospinal fluid; ^2^**RT-PCR from non-sterile samples**: upper respiratory tract samples (e.g., naso- or oropharyngeal swab or aspirate) or other non-sterile samples (e.g., stool).

**Table 2:** Intrapartum SARS-CoV-2 transmission (modified by WHO guidelines [23]).

INTRA- PARTUM Infection	(1) Maternal SARS-CoV-2 Infection	(2) Lack of In Utero Fetal SARS-CoV-2 Exposure (Birth–24 h)	(3) Intrapartum SARS-CoV-2 Exposure with Viral Persistence/Immune Response (24–48 h)
**Confirmed**	Suspect, probable or confirmed maternal infection **near the time of birth (14 days prior to 2 daysafter birth)**	**Negative** tests	**Positive RT-PCR** *(from a sterile sample or from repeated non-sterile samples at **2–7 days**)***OR** **Positive serology** (IgM or IgA) **at 7–14 days of age** *(confirmed by a second **positive** serology test **within 10 days** since the first one)*
**Possible**		**No** in utero fetal exposure tests performed	**Positive RT-PCR** *(from a sterile sample or from repeated non-sterile samples at **2–7 days**)* **OR** **Positive serology** (IgM or IgA) **at 7–14 days of age** *(confirmed by a second **positive** serology test **within 10 days** since the first one)*
**Unlikely**		**No** in utero fetal exposure tests performed	**Positive RT-PCR** *(from a sterile sample with a **negative** second sample at **2-7 days*** *or from a non-sterile sample with a **negative** second non-sterile sample* *at **2-7 days)*** **OR** **Positive serology** (IgM or IgA) **at 7–14 days of age** *(with a second **negative** serology test **within 10 days** since the first one)*

**Table 3:** Early Postnatal SARS-CoV-2 transmission (age > 48 h–28 days) (modified by WHO guidelines [23]).

POST- NATAL Infection	(1) Maternal SARS-CoV-2 Infection	(2) Lack of In Utero fetal SARS-CoV-2 Exposure (see above)	(3) Intrapartum SARS-CoV-2 Exposure with Viral Persistence/Immune Response (>48 h)
**Confirmed**	Suspected, probable or confirmed maternal infection **near the time of birth (14 days prior to 2 daysafter birth)**	**Negative** tests before 48 h of age	**Positive RT-PCR** at **age ≥48 h:** - **from a sterile sample** - **from non-sterile samples** *(with a second **positive** sample within 10 days since the first one)* **OR** **Positive serology** (IgM or IgA) **at age >14 days** *(confirmed by a second **positive** serology test **within 10 days** since the first one)*
**Possible**		**No** in utero/intrapartum exposure tests performed	**Positive RT-PCR** at ** age ≥48 h:** - **from a sterile sample** - **from non-sterile samples** *(with a second **positive** sample within 10 days since the first)* **OR** **Positive serology** (IgM or IgA) **at age >14 days** *(confirmed by a second **positive** serology test **within 10 days** since the first one)*
**Unlikely**		**No** in utero/intrapartum exposure tests performed	**Positive RT-PCR** at **age ≥48 h from a non-sterile sample** *(with a second **negative** sample within 10 days since the first one)* **OR** **Positive** serology (IgM or IgA) at **age >14 days** with a **negative** serology test **within 10 days** since the first one
**Indeterminate**		**No** in utero/intrapartum exposure tests performed	**Positive RT-PCR** at **age ≥48 h from a non-sterile sample** *(**without** a second corroboratory test)* **OR** **Positive** serology (IgM or IgA) **at age >14 days without** a second corroboratory test

**Table 4:** Distribution of clinical features of neonates with signs and symptoms of COVID-19. (modified from Raschetti et al., 2020 [22]).

Clinical Features	Neonates (%)
Respiratory manifestations (signs of respiratory distress, i.e., tachypnea, subcostal retractions and rhinitis)	51 (52.5%)
Fever	43 (44.3%)
Gastrointestinal manifestations (eating disorders, diarrhea, vomiting)	35 (36%)
Neurological manifestations (hypertonia/hypotonia, irritability/lethargy, apnea)	18 (18.6%)
Hemodynamic manifestations (tachycardia, hypotension)	10 (10.3%)
Other signs (conjunctivitis, hypothermia, cutaneous rash)	9 (9.2%)

## Data Availability

Not applicable.
